# A national survey of Chinese medicine doctors and clinical practice guidelines in China

**DOI:** 10.1186/s12906-017-1946-2

**Published:** 2017-09-06

**Authors:** Mengyu Liu, Chi Zhang, Qinglin Zha, Wei Yang, Ya Yuwen, Linda Zhong, Zhaoxiang Bian, Xuejie Han, Aiping Lu

**Affiliations:** 1 Institute of Basic Research in Clinical Medicine, China Academy of Chinese Medical Sciences, Nanxiaojie, Beijing 100700 China; 2 School of Chinese Medicine, Hong Kong Baptist University, Kowloon Tong, Kowloon 999077 Hong Kong; 3 School of Computer, Jiangxi University of Traditional Chinese Medicine, Nanchang, 330004 China

## Abstract

**Background:**

Clinical practice guidelines (CPGs) for Chinese medicine (CM) are being developed to assist doctors with appropriate decisions concerning CM care. To date, there has been little investigation on the perspectives of those to whom the guidelines are directed.

**Methods:**

A self-administered questionnaire was sent to 4503 doctors in 28 provinces of China in the latter half of 2012. Questions were organized around the topics of knowledge, application, practice changes, beliefs and outcomes of implementation. Basic classificatory data on specialties and years of qualification were also collected.

**Results:**

Replies were received from 4495 CM doctors (99.82%). Of these, 85.56% of CM doctors reported being familiar with CPG recommendations, but the overall adherence rate was only 50.39%. The length of time practicing CM may influence the rate of adherence, since 709 doctors (51.90%) with less than 5 years of experience reported never having followed CPGs. Doctors in nine specialties showed a modest degree of homogeneity in their attitudes towards CM diagnosis and treatment, which were generally positive. Most doctors regarded CPG-recommended therapies as safe (92%), economic (84%), and effective (76%). Approximately four-fifths of those questioned selected ‘acceptable’ (60.84%) and ‘acceptable after revision’ (19.23%) regarding their comprehensive assessment of the CPGs.

**Conclusions:**

An encouraging result from this survey is that the majority of CM doctors support the concept of CPGs for the practice of CM. However, the results of this survey also suggest that improving the adherence of CM doctors to the guidelines remains a major challenge to improving the practice standards for CM.

## Background

Clinical practice guidelines (CPGs) are classically defined as “systematically developed statements to assist doctors’ and patients’ decisions about appropriate healthcare for specific circumstances” and are designed to support decision-making processes during patient care [1, 2]. CPGs have the potential to influence the care delivered by doctors and the outcomes of patients [3]. Therefore, evidence on clinicians’ acceptance and implementation of CPGs is important. The majority of published studies on this topic have used cross-sectional surveys or medical record audits, exploring the gap between CPGs and doctors’ practices [4, 5].

Traditional medicine (TM) is speeding up the process of developing CPGs [6–9]. The World Health Organization (WHO) is keen on TM and has been active in the process of developing guidelines and standards for botanical medicine [10]. In China, the process of developing CPGs for Chinese medicine (CM) began approximately 30 years ago but has accelerated in recent years [11–14]. In 2008, the China Association of Chinese Medicine (CACM) released the ‘Guidelines for Diagnosis and Treatment of Common Internal Diseases in Chinese Medicine’ [15], which include 132 common internal diseases described by CM. These guidelines were developed by a national expert panel (comprising more than 200 experts) commissioned by the CACM. The CACM is a non-profit national academic organization in China that is an important social force in the development of Chinese medicine and acts as a link between the government and the professionals who practice Chinese medicine. Established in 1979, the CACM now includes the largest number of members from the Chinese medical professional community. The purpose of these guidelines is to “assist CM doctors in clinical decision making by describing a range of generally acceptable approaches”. These guidelines are still the most widely used guidelines in China [14].

Compared to Western medicine, clinical diagnoses and treatments in CM are less consistent, and the standards are poorer [16, 17]. Can the current CPGs for CM present conflicting priorities for doctors? Have traditional doctors accepted the CPGs recommendations on CM treatments? Despite the existence of doctor–guideline relationships, data on the extent of such relationships are sparse, and there has been no systematic exploration of doctor–guideline relationships since the first CM CPGs were issued. The perspectives of those who are being encouraged or recommended to use these guidelines in their everyday work are even less well understood. In this national survey of CM doctors’ knowledge, use and opinions of the CPGs (2008 edition), we were interested in exploring whether CM doctors accepted these guidelines, whether they followed them, and whether they considered that their practice had changed since they began following the guidelines.

## Methods

### Design and sites

This was a descriptive, cross-sectional study. Licensed CM doctors in 37 hospitals were chosen as the target population. Following the global emphasis on evidence-based quality care, the State Administration of Traditional Chinese Medicine of the People’s Republic of China (SATCM) selected 42 hospitals in China for pilot implementation of Chinese medicine clinical practice guidelines. These 42 hospitals are located in 31 provinces and municipalities throughout Mainland China. For this survey, 37 hospitals volunteered to provide data.

### Participants, recruitment and informed consent

Doctors (defined as currently licensed Chinese medicine doctors working in selected hospitals) were recruited using directories from each of the hospitals. Samples were obtained based on the doctors’ ID numbers included in the directories. Regional quotas were imposed on the samples, in line with the number of doctors affiliated with each of the hospitals. There are at least 100 participants from each hospital. If a doctor declined to complete the questionnaire, sampling continued until the required sample size was achieved. We first sent an introductory letter by standard mail to potential participants. The letter described the purpose of the survey and the procedures, indicating that a research team member would contact interested individuals within 1 week to invite them to participate and conduct the surveys. No incentive was offered to participants. Doctors were eligible to participate if they were currently practicing in their profession, practiced in one of the selected hospitals in China and were willing to anonymously complete the self-administered questionnaires. Doctors were screened to ensure that they were not working as participants in the development of CPGs for CM. In CM, the level of professional qualification may influence doctors’ views toward CPGs. Thus, in the current study, we stratified the samples depending on whether the doctors had 10 years, more than 10 years, or less than 10 years of professional qualification (greater than vs. less than or equal to 10 = 1:1). The Institutional Ethics Committee approved the study protocol [No 19, State Administration of Traditional Chinese Medicine of the People’s Republic of China 2012]. Written informed consent was obtained from all participants. The 3-page and 4-page questionnaires were sent to all selected doctors between July 1, 2012 and December 31, 2012.

### Questionnaire development and content

The process of questionnaire development involved systematic literature reviews and expert panel reviews, each of which contributed to the questionnaire’s face and content validity. To reduce social desirability bias, we tried to make the participants understand and value the research objectives of the study, including the detrimental consequences of providing incorrect information.

Each of the questionnaires consisted of two sections: a general and a CPG-specific section.

- Questionnaire I was developed to investigate doctors’ attitudes toward CPGs. The questionnaire was structured in a way that closely paralleled CM clinical guideline recommendations. The final questionnaire included mostly closed-end questions and a question with an open format. A section pertaining to CM doctor demographics was also added. Demographic information collected included years of qualification and specialty. The CPG-specific section concentrated on five key themes: (1) General attitude towards CPGs for CM; (2) Views on the quality of CPGs for CM; (3) A comprehensive assessment of CPGs for CM; (4) Coincidence with CPGs recommendations; and (5) The outcome of CPG applications. The coincidence with CPG recommendations means that the surveyed doctor agreed with the guidelines in terms of syndrome differentiation, CM therapeutic principles and the chosen method of treatment, such as the herbal formulas and CM health care approaches. A 4-point scale was used to rate the extent of agreement with the statements (ranging from 1. ‘Very poor’ to 4. ‘Very good’).
- Questionnaire II (each of the doctors completed at least 6 questionnaires by accessing the medical records of other doctors and ensuring that the selected records were not his/her own records) was developed to allow for direct comparison of CPG recommendations and clinical practice. CM doctors were asked about treating their patients by following CM CPGs, estimating their level of satisfaction after managing patients using CM CPGs, assessing the difference between CPG recommendations and their own records, and their level of compliance with these recommendations.

In our study of the responses to the statements regarding attitudes towards CPGs in general, we grouped the scores as 1 or 0 (Yes/No). Perceived approval ratings for each of the key CM diagnosis and treatment recommendations were determined by calculating the percentage of respondents that either agreed or strongly agreed (scores 3 and 4). The participants’ comprehensive assessments of CPGs for CM are given as the percentage responses of acceptable, acceptable after revision, completely acceptable and unacceptable. The overall acceptability score of CPGs for each specialty is calculated according to the following formula: Maximum possible score = 4 (strongly agree) × 4 (appraisers) = 16; Minimum possible score = 1 (strongly disagree) × 4 (appraisers) = 12. Regarding the perceived attitudes towards guidelines in general, we grouped scores of 3 and 4 (agree/strongly agree) as indicating agreement; a score of 2 as indicating some disagreement; and a score of 1 as indicating disagreement.

### Data collection and management

A statistician (WY) created an online database and instructed the research team members on the specifics of data entry. Team members researching every hospital entered the data accordingly, and upon completion, WY and another statistician (QLZ) conducted a full inspection and marked any illegible or ambiguous responses for reinvestigation (MYL). Questions that did not allow for multiple responses that were either left unmarked or were marked with two or more responses were considered missing data. The survey was conducted on a voluntary basis with agreement from the participants on the use of the collected data for scientific purposes.

### Statistical analysis

Using descriptive statistics, categorical data are presented as frequencies (%). Responses, demographic and professional characteristics of the doctors were allowed for in most categorical data, and ranking scores were collected and analyzed separately. Descriptive statistical analyses were performed with SAS version 9.4, (Cary, NC, USA: SAS Institute Inc.).

## Results

### Demographic characteristics

**Fig. 1:**
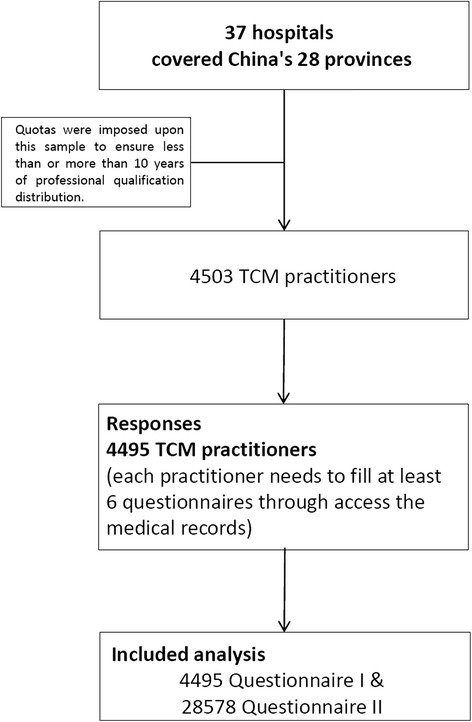
Flow chart for the survey in traditional Chinese medicine doctors

This study of CM doctors involving multi-specialties was accomplished via two self-administered questionnaires answered anonymously by doctors from 37 hospitals, covering 28 provinces in mainland China (Fig. 6 in Appendix). In total, 4495 eligible Questionnaires I and 28,578 Questionnaires II were returned. The flowchart depicts the survey decision process (Fig. 1). The general characteristics of the respondents are listed in Table 1. Since the samples are stratified based on whether the participants have more or less than 10 years of experience, half of the participants (51%, *n* = 2245) have 10 years of professional qualification; similarly, half of the participants (50.83%, *n* = 2885) are doctors who specialize in internal medicine.

**Table 1:** Characteristics of 4495 survey respondents

Characteristic	Total no.	Percentage
Professional		
≤ 5 years	1369	30.46
6–10 years	876	19.49
11–20 years	1114	24.78
21–30 years	910	20.24
> 30 years	145	3.22
Not reported	81	1.80
Specialty		
Internal medicine	2885	64.18
Oncology	165	3.67
Surgery	83	1.85
Gynaecology	350	7.79
Pediatrics	435	9.68
Ophthalmology	40	0.89
Otolaryngology	151	3.36
Anorectal	263	5.85
Dermatology	123	2.74

*Abbreviations*: *CPG* clinical practice guideline, *CM* Chinese medicine; *WHO* World Health Organization

### General attitude towards CPGs for CM

**Fig. 2:**
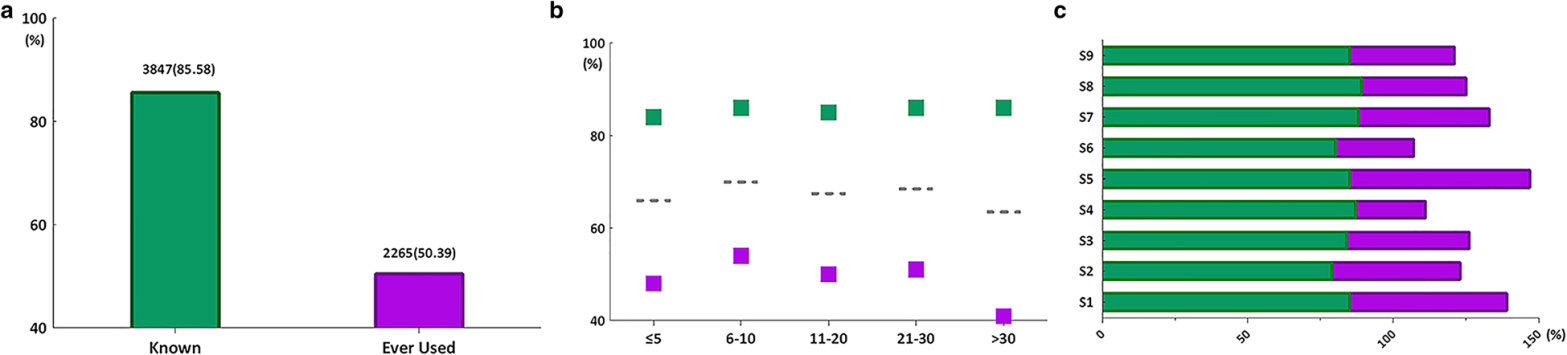
General attitude towards clinical practice guidelines for traditional Chinese medicine. **a** Known in green means the participant was familiarized with contents of clinical practice guidelines (CPGs) for traditional Chinese medicine (CM). Ever used in purple, means the participant used CPGs in his/her clinical practice. **b** The two columns of the figure show the total number and percentage of CM doctors known (column 1) and the number and percentage of reported ever used CPGs (column 2); **c** Views of CM doctors on CPGs by nine specialties. S1: Internal medicine; S2: Oncology; S3: Surgery; S4: Gynaecology; S5: Paediatrics; S6: Ophthalmology; S7: Otolaryngology; S8: Anorectal; S9: Dermatology

A majority (85.56%) of respondents stated that they were familiar with CPGs; however, significantly fewer (50.39%) claimed to be following some form of CPGs (Fig. 2-a). The level of experience was associated with whether doctors followed guidelines. The data indicated that middle-aged doctors were more likely to follow the guidelines. The percentage of doctors that were familiar with/reported following CPGs had experience in the range of 21–30 years (86.70%/51.76%, respectively), 11–20 years (85.55%/50.99%, respectively) 6–10 years (86.07%/54.20%, respectively) and less than 5 years of professional experience (84.41%/48.10%, respectively). The percentage of participants who were familiar with or reported following CPGs also varied depending on different specialties: 85.06% and 62.76% among pediatricians, respectively; 85.44% and 54.70% among internal medicine doctors, respectively; 88.74% and 45.70% among otolaryngologists, respectively; 79.39% and 44.24% among oncologists, respectively; 84.34% and 42.17% among surgeons, respectively; 85.37% and 36.59% among dermatologists, respectively; 89.35% and 36.12% among proctologists, respectively; 80.00% and 27.50% among ophthalmologists, respectively; and 87.14% and 24.57% among gynecologists, respectively.

### Views on the quality of CPGs for CM

**Fig. 3:**
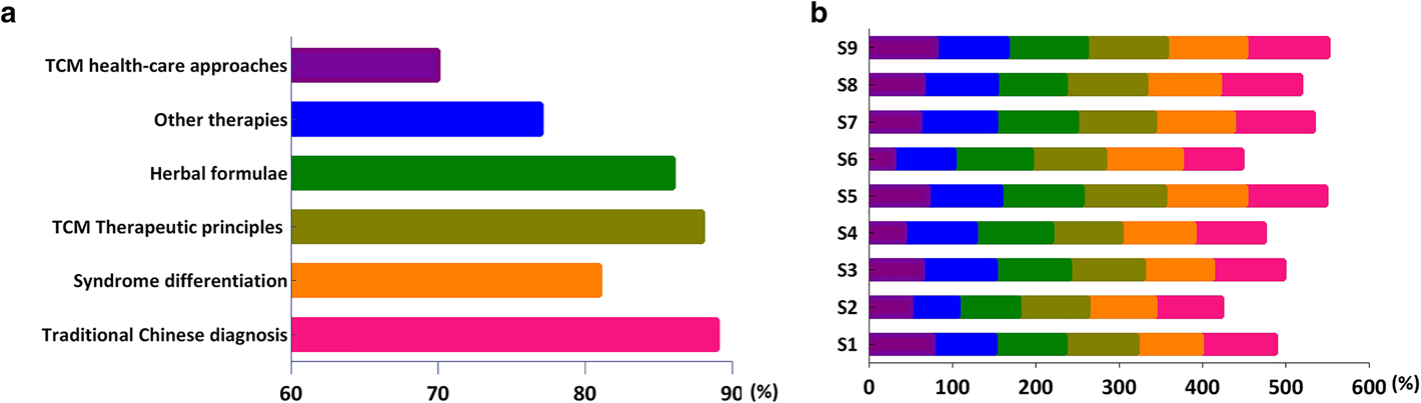
Attitudes towards various Chinese medicine items of clinical practice guidelines (%). **a** Attitudes towards six Chinese medicine items of CPGs. Six items include traditional Chinese diagnosis (*pink*), syndrome differentiation (*orange*), CM therapeutic principles (*olive*), herbal formulae (*green*), other therapies (*blue*) and CM health-care approaches (*purple*). Perceived approval rating for each of the key CM diagnosis and treatment recommendations were determined by calculating the percentage of respondents that either agreed or strongly agreed (score 3 and 4). The following are some of the characteristics of CM: • syndrome differentiation (辨證) the process of overall analysis of clinical data to determine the location, cause and nature of a patient’s disease and achieving a diagnosis of a pattern/syndrome, also called pattern differentiation. • therapeutic principle & method of treatment (治則治法) a general rule that should be followed in treating disease and any specific intervention derived from a principle of treatment. • formula (方劑) prescription, recipe. • CM health-care approaches (養生) traditional health-care to promote health, prevent disease and enhance longevity, also called health preservation/cultivation. **b** Attitudes towards six Chinese medicine items of CPGs by nine specialties. S1: Internal medicine; S2: Oncology; S3: Surgery; S4: Gynaecology; S5: Paediatrics; S6: Ophthalmology; S7: Otolaryngology; S8: Anorectal; S9: Dermatology

Overall, 93% of doctors believed that CPGs were valuable for the prevention and treatment of diseases, had the proper ‘scope and purpose’, and used ‘professional terminology’. Most doctors agreed with the content of common guidelines for traditional Chinese diagnostics (89.68%), syndrome differentiation (81.96%), CM therapeutic principles & methods of treatment (88.34%), herbal formulations (86.1%), complimentary therapies (77.91%), and CM health care approaches (70.75%) (Fig. 3-a). Of these, proctologists (96.96%) and pediatricians (96.32%) viewed diagnostic guidelines more favorably. Internal medicine doctors (77.09%) showed the highest positive attitudes toward CM syndrome differentiation. Almost all pediatricians (99.31%) agreed with the CM therapeutic principles & methods of treatment. Both oncologists (72.73%) and proctologists (72.73%) showed the lowest level of agreement with herbal formulations. Doctors differed widely in their opinions about CM health care approaches (from 30.00% to 80.49%) (Fig. 3-b).

### Comprehensive assessment of CPGs for CM

**Fig. 4:**
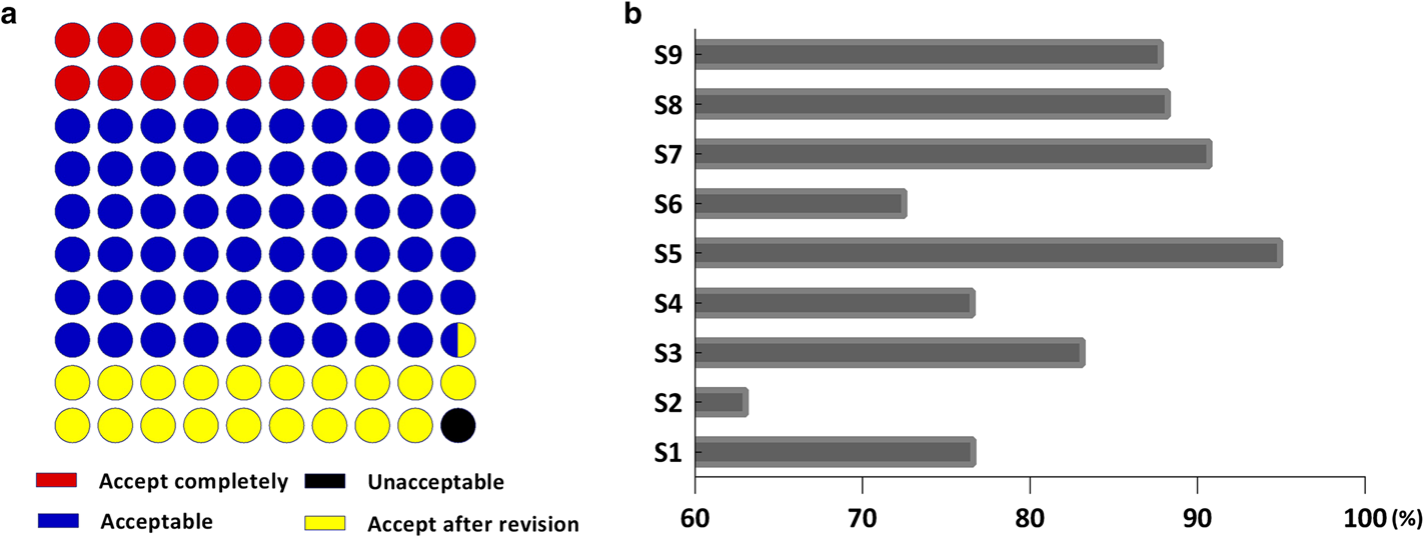
Views on comprehensive assessment of clinical practice guidelines for traditional Chinese medicine. **a** Comprehensive assessment requires CM clinicians to make a judgment of CPGs. Perceived views on comprehensive assessment of CPGs for CM are given as percentage response rates of accept completely (*red*), acceptable (*blue*), accept after revision (*yellow*) and unacceptable (*blue*). Four-fifths (80.07%) selected ‘acceptable’ (60.84%) and ‘accept after revision’ (19.23%), ‘accept completely’ (18.81%) and ‘unacceptable’ (1.12%). **b** Comprehensive assessment of CPGs for CM by nine specialties. S1: Internal medicine; S2: Oncology; S3: Surgery; S4: Gynaecology; S5: Paediatrics; S6: Ophthalmology; S7: Otolaryngology; S8: Anorectal; S9: Dermatology. S1 (76.60%), S2 (63.03%), S3 (83.13%), S4 (76.57%), S5 (94.94%), S6 (72.5%), S7 (90.73%), S8 (88.21%) and S9 (87.8%)

Approximately four-fifths (80.07%) of participants selected either ‘acceptable’/‘completely acceptable’ (60.84%) or ‘Acceptable after revision’ (19.23%) concerning a comprehensive assessment of the CPGs for CM, whereas 18.81% selected ‘completely acceptable and 1.12% chose ‘unacceptable’ (Fig. 4-a). In descending order, the comprehensive assessment scores among different specialties were as follows: pediatrics (94.94%), otolaryngology (90.73%), anorectal medicine (88.21%), dermatology (87.8%), surgery (83.13%), internal medicine (76.60%), gynecology (76.57%), ophthalmology (72.5%), and oncology (63.03%) (Fig. 4-b).

### Coincidence with CPG recommendations

**Fig. 5:**
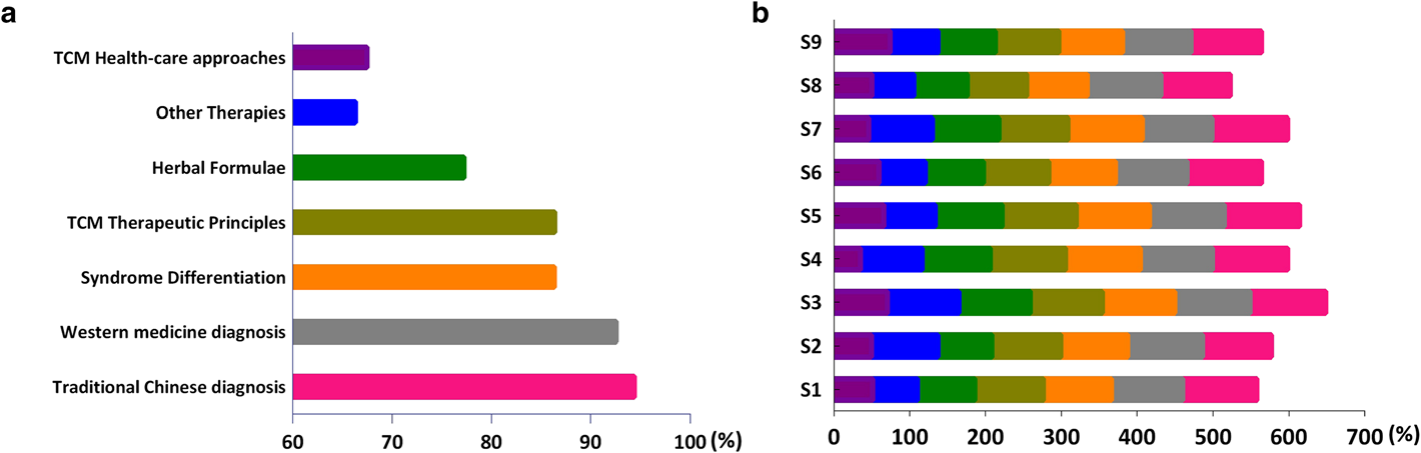
The Coincidence of clinical practice guidelines for traditional Chinese medicine recommendations and doctor individual interests. **a** The Coincidence of CPGs recommendations. Seven items include traditional Chinese diagnosis (*pink*), western medicine diagnosis (*grey*), syndrome differentiation (*orange*), CM therapeutic principles (*olive*), herbal formulae (*green*), other therapies (*blue*) and CM health-care approaches (*purple*). Perceived approval rating for each of the key CM diagnosis and treatment recommendations were determined by calculating the percentage of respondents that either agreed or strongly agreed (score 3 and 4). **b** The Coincidence of CPGs recommendations and nine specialties. S1: Internal medicine; S2: Oncology; S3: Surgery; S4: Gynaecology; S5: Paediatrics; S6: Ophthalmology; S7: Otolaryngology; S8: Anorectal; S9: Dermatology

**Fig. 6:**
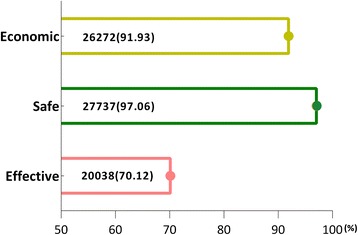
National survey map of 2012 in China: traditional Chinese medicine doctors and clinical practice guidelines

The overall level of coincidence with traditional Chinese diagnostics (94.38%) was slightly higher than with Western medicine diagnostics (92.56%). The following percentage of doctors agreed with the different categories: syndrome differentiation (86.32%), CM therapeutic principles & methods of treatment (86.36%), herbal formulas (86.1%), and CM health care approaches (67.49%). The level of agreement with complimentary therapies (66.33%) was the lowest overall of the six surveyed items (Fig. 5-a). The degree of coincidence with the section ‘CM diagnosis’ was almost complete for the following specialties: otolaryngology (99.9%), surgery (99.62%), pediatrics (99.12%) and ophthalmology (99.08%). However, other specialties showed coincidences above 90%. Compared with other specialties, internal medicine (90.49%) and surgery (92.2%) showed low rates of coincidence in the section ‘Western medicine diagnosis’. Ophthalmology (98.71%) and surgery (97.83%) showed higher rates of coincidence in terms of syndrome differentiation compared to other specialties, whereas oncology (79.51%) and internal medicine (83.61%) ranked relatively lower than other specialties. Oncology ranked lowest in terms of both CM therapeutic principles & methods of treatment (78.57%) and herbal formulations (70.61%). Ophthalmology (34.87%) and surgery (45.65%) showed unacceptably low coincidence rates with CM health care approaches. However, even the specialty that scored the highest, internal medicine (73.8%), did not have a much higher rate than the rates of ophthalmology and surgery (Fig. 5-b).

### The outcomes of following CPGs

The outcomes of following CPGs were surveyed in terms of their perceived effectiveness, safety and cost. Most doctors regarded CPG-recommended therapies as safe (92%), economic (84%), and effective (76%). Questionnaire II identified a total of 20,038 cases (70.12%) that considered the CPG recommendations as effective. The level of agreement with the guidelines in terms of safety was similar to that of their perceived cost. The total number of cases reporting good safety outcomes was 27,737 (97.06%), and the total number of patients with improved cost-effective outcomes was 26,272 (91.93%). Pediatricians reported the highest levels of effectiveness (95.16%) and safety (95.85%). The results showed that these CPGs for CM obtained good scores in terms of efficacy, safety and cost-effectiveness from CM doctors’ points of view.

## Discussion

This national survey demonstrated that CM doctors have a positive attitude towards the national CPGs for CM. In addition, only half of the participants adhered to the CPG recommendations. However, most doctors thought the current CPGs were “very useful” or “useful” for improving the safety, efficacy and cost-effectiveness of their patients’ treatments.

### Strengths

In China, to the best of our knowledge, this study is the first to explore the perceptions, beliefs and attitudes of CM clinicians. China Health Statistics (2012) indicated that there were approximate 26.7 hundred thousand registered CM doctors in China [18]. The 4495 CM doctors surveyed in this study accounted for 1.7% of the whole CM doctor population in China. In addition, this survey covered China’s 28 provinces (excluding Inner Mongolia, Qinghai, the Tibetan autonomous region, Hong Kong and Macao Special Administrative Regions, and Taiwan).

In general, CM doctors in our study were familiar with CPGs. It is important to note that the positive attitude towards CPGs found in our sample of CM doctors may be related to the fact that all CM doctors were affiliated with CPG dissemination project hospitals. Certainly, this could be interpreted as a bias, since it can result in strong positive attitudes among the target group. While recognizing the potential benefits of practice guidelines, the overall adherence rate (50.39%) reported by CM doctors was much lower than their familiarity (85.56%) with the guideline recommendations. We further uncovered that the rates of adherence with the recommendations varied depending on clinical experience. For doctors with less than 5 years of qualification, the rate of CPG adherence was only 48.10%. An educational opportunity exists to encourage young doctors to follow CPGs and improve the quality of care. Many factors may influence the effective implementation of CPGs in practice. Barriers to guideline adherence can be related to the individual patient, the organizational context, and the social and cultural context of the healthcare system [19]. Although these guidelines are widely available, clinical diagnoses and treatments in CM are less consistent; specifically, the strong clinical evidence and the standards are poorer, which can have a negative impact on the adherence of doctors to these recommendations. A qualitative analysis of the open questions included in our survey lead to a common suggestion: that CM recommendations may be further classified at the system and individual level. This conforms to the fundamental concepts of CM. Obviously, an adequate analysis of the barriers that prevent CM doctors from following CPGs in practice need to be demonstrated by further studies to improve guideline adherence.

To our surprise, after assessing 28,578 medical records, there was almost no difference in terms of general coincidence between traditional Chinese diagnosis (94.38%) and Western medicine diagnosis (92.56%). This lack of difference may be related to the fact that in recent decades, CM doctors in China received both CM and Western medicine training as well as continuous medical education. Moreover, from the points of view of 4495 CM doctors, the contents of the core elements of CM practice were optimal, and they agreed with the content of the common guidelines related to syndrome differentiation (81.96%), CM therapeutic principles & methods of treatment (88.34%), and herbal formulations (86.1%); correspondingly, the coincidences were 86.32%, 86.36% and 86.1%, respectively, when analyzing the patients’ records. Based on these results, we have reason to believe that there are no irreconcilable differences between the general CPGs for CM and the personal practices of CM doctors, when each is held in its proper place.

Our study also showed that the degree of familiarity and incorporation of CPGs in CM practice differed by specialty. Pediatricians were more aware of the national guidelines for CM treatments (85.06%) than doctors practicing other specialties, and they reported a higher rate of incorporation of these guidelines into their practice (62.76%) and higher levels of effectiveness (95.16%) and safety (95.85%) after incorporating these guidelines. In contrast, oncology specialists scored relatively lower than other doctors in terms of syndrome differentiation (79.51%), CM therapeutic principles & methods of treatment (78.57%) and herbal formulations (70.61%). These differences are due to variations in the internal characteristics of these specialties [20]. Furthermore, the existence of different conditions with different prognoses could be another reason explaining the different views of pediatricians and oncologists on CPGs. Due to these contrasts, it is essential to have these differences in mind for the future development of CPGs.

Information gathered from the survey shows there is a need to harmonize CM health care approaches in the CPGs. A striking finding in our study was the low level of recognition (70.75%) that recommendations on CM health care approaches would benefit patients, showing the lowest adherence rate (67.49%) based on the patients’ records. In CM, traditional healthcare to promote health, prevent disease and enhance longevity is also called health preservation/cultivation [21]. This diversified approach has become a feature of CM health care; consequently, the low adherence rate can be recognized.

### Limitations

Our study has some potential limitations. As with most surveys, the main weakness of this study is the generalizability and reliability of CM doctors’ responses. These may be affected by various parameters. First, this is a self-reported survey. Although the questionnaire was anonymous, respondents may be tempted to idealize their practice. The second questionnaire asking the CM doctor to rate other doctors’ medical records in terms of compliance with CPG, may lead to social desirability bias. Second, the high response rate (99.82%) made it impossible to rule out selection bias and to know whether respondents differ from non-respondents. Third, due to the lack of other studies focusing on doctors’ attitudes towards CPGs for CM or TM, we cannot draw a parallel between this study and others. In general, a high response rate is likely to reduce bias; however, it is important not to overlook other types of bias which cannot be overcome with a high response rate. In this case, even a response rate of 99.82% would not guarantee that the results were free of bias because the data collection was confined to a network of hospitals selected as standardized CM treatment demonstration sites. Despite such limitations, a good number of responses were collected across 28 provinces in mainland China, and the findings are important and relevant even if they are not entirely generalizable.

Our previous study indicated that only approximately 60% of CM clinical studies used CPGs as their clinical assessment criteria [22]. With the results of this survey, it seems that improved education of doctors, together with more time spent by doctors training on CPGs and improved financing for CM CPGs training programs, as well as clear and strong cultural and institutional changes in favor of evidence-based CM practice, would be the best way to improve adherence to CM CPGs.

## Conclusion

In conclusion, an encouraging observation from this survey is that the majority of doctors support the concept of CM CPGs. However, this survey also showed that in reality, only half of the doctors follow CPGs. Marked differences exist in the degree of acceptance and implementation of CPGs between doctors practicing nine different specialties. Further research is merited to find effective strategies that may improve the implementation and application of CM CPGs.
